# Changes in Characteristics and Outcomes of Patients Undergoing Surgery for Hip Fractures Following the Initiation of Orthogeriatric Service: Temporal Trend Analysis

**DOI:** 10.1007/s00223-021-00906-4

**Published:** 2021-08-27

**Authors:** Ben Fluck, Keefai Yeong, Radcliffe Lisk, Hazel Watters, Jonathan Robin, David Fluck, Christopher H. Fry, Thang S. Han

**Affiliations:** 1 Institute of Cardiovascular Research, Royal Holloway, University of London, Egham, TW20 0EX Surrey UK; 2 Department of Orthogeriatrics, Ashford and St Peter’s NHS Foundation Trust, Guildford Road, Chertsey, KT16 0PZ Surrey UK; 3 Department of Acute Medicine, Ashford and St Peter’s NHS Foundation Trust, Guildford Road, Chertsey, KT16 0PZ Surrey UK; 4 Department of Cardiology, Ashford and St Peter’s NHS Foundation Trust, Guildford Road, Chertsey, KT16 0PZ Surrey UK; 5 School of Physiology, Pharmacology and Neuroscience, University of Bristol, Bristol, BS8 1TD UK

**Keywords:** Mortality, Length of stay, Discharge destination, Join-point analysis, Temporal trends

## Abstract

The Blue Book published by the British Orthopaedic Association and British Geriatrics Society, together with the introduction of National Hip Fracture Database Audit and Best Practice Tariff, have been influential in improving hip fracture care. We examined ten-year (2009–2019) changes in hip fracture outcomes after establishing an orthogeriatric service based on these initiatives, in 1081 men and 2891 women (mean age = 83.5 ± 9.1 years). Temporal trends in the annual percentage change (APC) of outcomes were identified using the Joinpoint Regression Program v4.7.0.0. The proportions of patients operated beyond 36 h of admission fell sharply during the first two years: APC =  − 53.7% (95% CI − 68.3, − 5.2, *P* = 0.003), followed by a small rise thereafter: APC = 5.8% (95% CI 0.5, 11.3, *P* = 0.036). Hip surgery increased progressively in patients > 90 years old: APC = 3.3 (95% CI 1.0, 5.8, *P* = 0.011) and those with American Society of Anaesthesiologists grade ≥ 3: APC = 12.4 (95% CI 8.8, 16.1, *P* < 0.001). There was a significant decline in pressure ulcers amongst patients < 90 years old: APC =  − 17.9 (95% CI − 32.7, 0.0, *P* = 0.050) and also a significant decline in mortality amongst those > 90 years old: APC =  − 7.1 (95% CI − 12.6, − 1.3, *P* = 0.024). Prolonged length of stay (> 23 days) declined from 2013: APC =  − 24.6% (95% CI − 31.2, − 17.4, *P* < 0.001). New discharge to nursing care declined moderately over 2009–2016 (APC =  − 10.6, 95% CI − 17.2, − 2.7, *P* = 0.017) and sharply thereafter (APC =  − 47.5%, 95%CI − 71.7, − 2.7, *P* = 0.043). The rate of patients returning home was decreasing (APC =  − 2.9, 95% CI − 5.1, − 0.7, *P* = 0.016), whilst new discharge to rehabilitation was increasing (APC = 8.4, 95% CI 4.0, 13.0; *P* = 0.002). In conclusion, the establishment of an orthogeriatric service was associated with a reduction of elapsed time to hip surgery, a progressive increase in surgery carried out on high-risk adults and a decline in adverse outcomes.

**Supplementary Information:**

The online version contains supplementary material available at 10.1007/s00223-021-00906-4.

## Introduction

Hip fracture is a common condition in older people, sharing the largest proportion of hospital admissions amongst all types of fractures [1]. Patients with a hip fracture have increased risk of many adverse outcomes in hospital including prolonged hospitalisation, malnutrition, pressure ulcers, mortality and requirement for higher levels of care [2–6]. Consequently, the personal and social costs of hip fractures are enormous [7, 8]. Whilst surgery is the main treatment to fix hip fractures, post-operative patient-care plays a key role in functional recovery, minimising hospital-associated complications and timely discharge. Efforts have been made to improve hip fracture management over the past 15 years. These include publication in 2005 of the Blue Book jointly sponsored by the British Orthopaedic Association and the British Geriatrics Society [9], providing guidance for the care of patients with fragility fractures. This was followed by the launch of a national audit in 2007 for England and Wales [10]. This programme included a web-based audit tool, the National Hip Fracture Database (NHFD), which allows hospitals to monitor the quality and outcomes of care provided to the individual patient. This led to the introduction of the pay-for-performance initiative (the Best Practice Tariff for hip fractures) in 2010, which financially incentivises individual hospitals by paying them a supplement for each patient whose care satisfied six clinical standards, including surgery within 36 h of arrival [11].

A number of studies have examined the effect of orthogeriatric care on outcomes of hip fracture patients. These studies were based on relatively small number of patients [12–15], short periods of observation [12, 13, 16], relatively few outcomes and limited information on patient characteristics [12–16]. In this study of patients undergoing hip fracture surgery at our centre, we have examined temporal trends over the past decade in patient characteristics, post-operative outcomes and discharge destination.

## Methods

### Milestones of Service Development

Prior to 2010, hip fracture care was led solely by the orthopaedic team. Patients were referred to general medical teams only on an ad hoc basis when deemed necessary. In September 2010, Ashford and St Peter’s NHS Foundation Trust initiated an orthogeriatric service for joint care of hip fracture patients, led by two newly appointed orthogeriatricians. These consultants redesigned the service based on the Lean principles, locally known as EQuIP (efficiency, quality, innovation and productivity programme). The aim was to target key areas for service improvement to i) achieve the Best Practice Tariff including elapsed time to surgery within 36 h of arrival; ii) achieve the standards of care for hip fracture patients as set out in the Blue Book; iii) set up facilitated care pathways for these patients, notably prioritisation to theatre and the integration of the orthogeriatricians into the routine care for hip fracture; iv) improve patient outcome and experience and v) improve hospital length of stay (LOS). At this point, hip fracture patients were cared within the geriatric ward with the orthopaedic surgeon acting as a consultative specialist, whilst the orthogeriatrician was responsible for the care of the patients. There were two consultant ward rounds a week during this period [17]. The next change was in 2013 when the orthogeriatric supportive discharge (OSD) team was set up. This was a targeted intervention to reduce the hospital LOS. By 2016, daily ward rounds led by a consultant of the week (the COW model for orthogeriatrics and orthopaedics) were added to the service to support continuity of care. The COW model is an integrated shared-care model [15] involving a seven days-a-week ward round led by an orthopaedic surgeon and also including an orthogeriatrician on Monday–Friday. Both specialists worked closely with a multi-disciplinary team of physiotherapists, nurses, occupational therapists and social service workers. Before each ward round, a board round and a multidisciplinary team meeting was held, led by both specialists, or only the orthopaedic surgeon at weekends [18].

### Study Design, Participants and Setting

We conducted a cross-sectional study of older individuals admitted with hip fractures to a National Health Service hospital, serving a catchment population of over 410,000 people.

### Measurements

Through our participation in the NHFD Audit Programme [4, 19, 20], data from time of admission to discharge were prospectively collected by a Trauma Coordinator for all patients admitted with a hip fracture. Data comprised demographic and care quality measures including age, sex, mobility within one day after hip surgery, pressure ulcers, LOS and mortality in hospital, as well as discharge destination. Data were collected from 2009 to 2019 and routinely updated and checked by the orthogeriatrician to ensure completeness and accuracy. The American Society of Anesthesiologists (ASA) classification was used to assess the physical status of patients [21].

### Categorisation of Variables

Mobilisation within one day after hip operation was defined as those who could start rehabilitation no later than the day after surgery [22]; prolonged LOS as a LOS > 23 days in hospital (the upper quartile of LOS); change in discharge destination as those who came from their own home before admission but transferred to places where increased care was provided, including rehabilitation units, residential home or nursing care. Categorisation of ASA was examined in patients with grade 3 (severe systemic disease) and grade 4 (severe systemic disease that is a constant threat to life), as well as grade ≥ 3. Delay in elapsed time to surgery was considered if hip surgery was beyond 36 h from time of admission as defined by the Best Practice Tariff criteria [11].

### Statistical Analysis

Characteristics of patients were assessed by descriptive statistics using IBM SPSS Statistics, v25 (IBM Corp., Armonk, NY). Temporal trends in outcome measures over time were identified using the Joinpoint Regression Program 4.7.0.0 [23]. This technique detects join points in data sets and calculates the annual percentage change (APC) for individual linear segments (i.e. different slopes) when one or more join points exist, as well as an average APC (AAPC) for the entire period of study. If join points do not exist, then APC and AAPC are the same.

## Results

### General Description Over the Period of the Survey

**Fig. 1:**
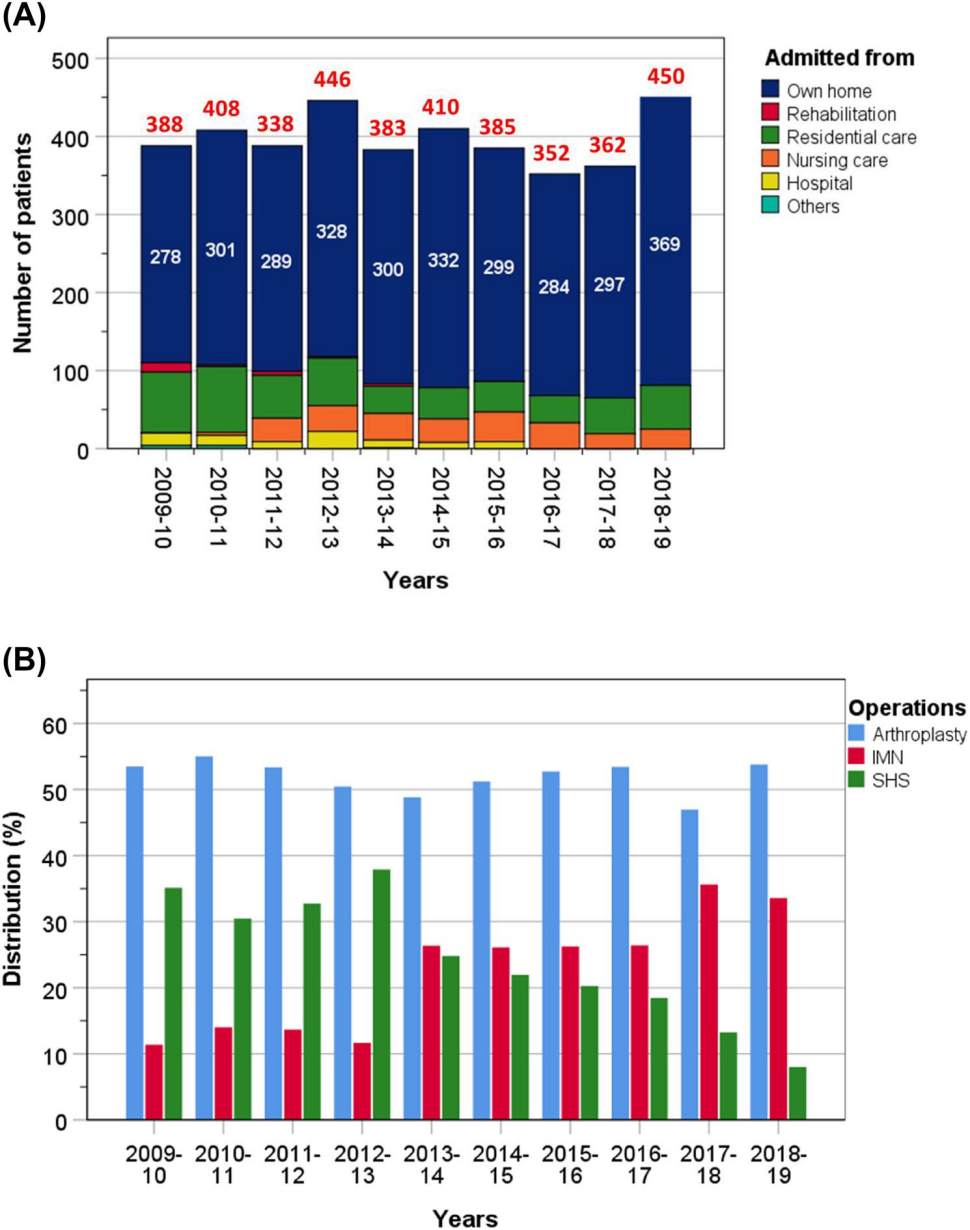
Annual number of patients admitted to hospital with a hip fracture between 2009 and 2019. Numbers of patients admitted from their own home are in white font and the total annual numbers are in red font (**A**); annual distribution of operative management of hip fractures (**B**)

Data from a total of 3972 patients (1081 men, 27.2%) and (2891 women, 72.8%) were available for analysis, with a mean age of 83.5 ± 9.1 years (median 84.9 years, IQR = 79.0–89.9), with a quarter of patients older than 90 years. Patients were analysed for all outcomes, except for the analysis of discharge destination where patients had to be from their own home originally and survived to discharge. Overall, there were 3077 patients (77.5%) who came from their own home, 24 (0.6%) from rehabilitation units, 529 (13.3%) from residential care, 246 (6.2%) from nursing care, 87 (2.2%) from hospital and 9 (0.2%) from other types of residence (Fig. 1).

The median elapsed time to surgery was 21.8 h (IQR = 16.8–29.8). Overall, 61.7%, 17.8% and 20.5% were operated within 24 h, 24–36 h and > 36 h, respectively (see also Table 1). Of these, 42.0% had ASA grade 3 and 5.9% with ASA grade 4, so that 48.0% had ASA grade ≥ 3 (including three with ASA grade 5; see Table 1). The median LOS during admission was 13.3 days (IQR = 8.3–23.3). The majority of patients received arthroplasty (51.9%), followed by similar proportions of treatment with an intramedullary nail (IMN) (22.4%) or a sliding hip screw (SHS) (24.4%); a small proportion received other techniques such as hybrid total hip replacement (1.3%). The majority of anaesthesia used spinal block with general anaesthetics (42.2%) or spinal block only (18.3%), whilst just over a quarter used general anaesthetics only.

**Table 1:** Outcomes of 3972 patients undergoing surgery for a hip fracture

	n	%
*Elapsed time to surgery*		
≥ 36 h	814	20.5
Age > 90 years at operation (top quartile of age)	993	25.0
*ASA classification*		
Grade 3	1666	42.0
Grade 4	236	5.9
Grade ≥ 3 (including 3 patients with grade 5)	1905	48.0
*Anaesthesia types*		
Spinal block with general anaesthetics	1543	42.2
Spinal block only	668	18.3
General anaesthetics only	919	25.1
Others	526	14.4
*Surgical techniques*		
Arthroplasty	2063	51.9
IMN	888	22.4
SHS	968	24.4
THR hybrid and others	51	1.3
*Post-operative outcomes*		
Failure to mobilise within one day of hip surgery	398	10.0
Hospital-acquired pressure ulcers	132	3.3
Mortality in hospital	225	5.7
Prolonged LOS in hospital (> 23 days)^†^	991	25.0
*Discharge destinations amongst patients who were admitted from their own home (n = 3077)*		
Returned back home	1822	59.2
New discharge to rehabilitation	772	25.1
New discharge to residential care	66	2.1
New discharge to nursing care	138	4.5
Other destinations	123	4.0
*Antiresorptive therapy*		
Antiresorptive therapy on admission	183	4.6
Newly prescribed antiresorptive therapy	3296	83.0
No antiresorptive therapy on discharge	485	12.3

^†^Top quartile of LOS

After hip surgery, 10% of patients failed to mobilise within one day, 3.3% developed a pressure ulcer, 5.7% died in hospital and 25.0% of patients stayed longer than 23 days in hospital. Amongst patients who were admitted from their own home, 59.2% returned back home, 25.1% were newly discharged to rehabilitation, 2.1% to residential care, 4.5% to nursing care and 4.0% to other residence. Antiresorptive therapy was used in only 4.6% of patients on admission and was prescribed in 83% of patients by discharge (Table 1). The majority of whom were prescribed oral anti-resorptive agents (93.5%), whilst only 6.5% received an injectable agent (denosumab). None of the patients were prescribed with anabolic agents such teriparatide or parathyroid hormone.

### Trends in Perioperative Characteristics and Operative Outcomes

**Fig. 2:**
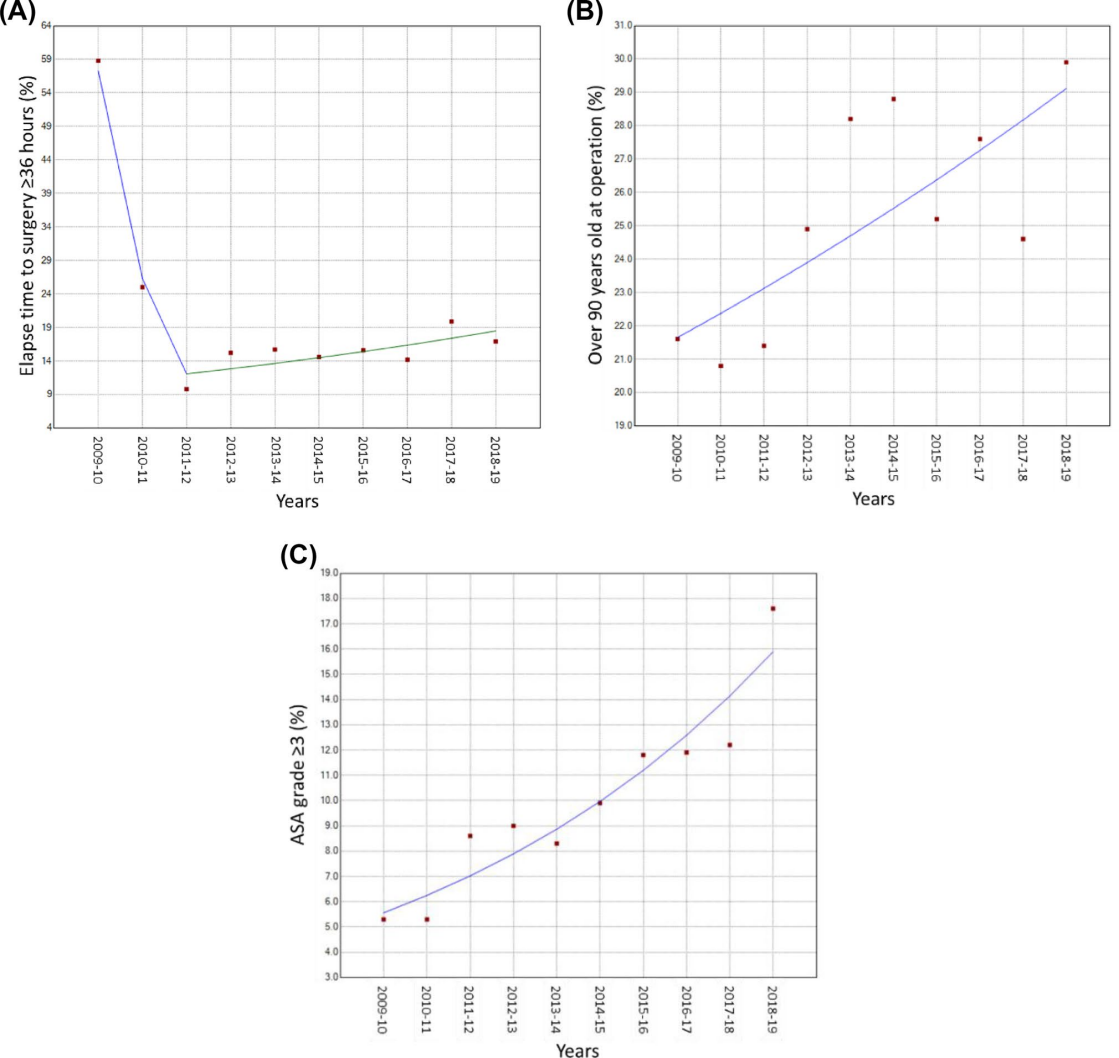
Temporal trends in elapsed time to surgery beyond 36 h (**A**), hip surgery for patients older than 90 years (**B**) and those with ASA classification ≥ 3 (**C**)

The proportions of patients operated beyond 36 h fell sharply during the first two years (2009–2011): − 54.1% (95% CI  − 68.7, − 32.6, * P* = 0.003), followed by a small rise thereafter: APC = 6.3% (95% CI 0.9, 11.9, * P* = 0.029) (Fig. 2A, Table 2). During the first two years, the mean age at operation was lowest at about 82.5 years, rising gradually to a plateau of about 84 years four years later (Supplement Fig. 1). During the period of study, hip operation for patients > 90 years rose significantly (APC = 3.3%, 95% CI 1.0, 5.8, * P* = 0.011) (Fig. 2B) and those with ASA grade ≥ 3: APC = 12.4% (95% CI 8.8, 16.1, * P* < 0.001) (Fig. 2C). Over this time, the use of arthroplasty did not change, but there was an increased use of IMN: APC = 14.6% (95% CI 9.1, 20.3, * P* < 0.001) and a decrease of SHS: APC =  − 13.5% (95% CI − 18.1, − 6.2, *P* < 0.001) (Supplement Fig. 2). There were no changes in patient numbers coming from nursing care.

**Table 2:** Annual percentage change for amongst patients undergoing operation for hip fractures

	APC (%)	95% CI	*P*
Elapsed time beyond ≥ 36 h (2009–2011)	− 54.1	− 68.7, − 32.6	0.003
Elapsed time beyond ≥ 36 h (2011–2019)	6.3	0.9, 11.9	0.029
Age at operation > 90 years	3.3	1.0, 5.8	0.011
ASA grade 3	10.4	6.7, 14.2	< 0.001
ASA grade 4	30.8	22.1, 40.0	< 0.001
ASA grade ≥ 3	12.4	8.8, 16.1	< 0.001
Arthroplasty	− 0.5	− 1.8, 0.8	0.383
IMN	14.6	9.1, 20.3	< 0.001
SHS	− 13.5	− 18.1, − 6.2	< 0.001
*Post-operative outcomes (n = 3972)*			
Failure to mobilise within 1 day of surgery	2.9	− 8.3, 15.4	0.494
Hospital-acquired pressure ulcers (all)	− 12.5	− 24.2, 1.1	0.065
Hospital-acquired pressure ulcers (< 90 yrs)	− 17.9	− 32.7, 0.0	0.050
Hospital-acquired pressure ulcers (> 90 yrs)	− 8.4	− 18.0, 2.3	0.105
Mortality in hospital (all)	− 2.0	− 6.2, 2.4	0.318
Mortality in hospital (< 90 yrs)	1.0	− 7.2, 9.8	0.797
Mortality in hospital (> 90 yrs)	− 7.1	− 12.6, − 1.3	0.024
LOS in hospital > 23 days (2009–2013)	− 1.0	− 23.4, 29.5	0.927
LOS in hospital > 23 days (2013–2018)	− 24.4	− 37.4, − 8.5	0.013
*Discharge destinations (n = 3077)*			
New discharge to nursing care (2009–2016)	− 10.6	− 17.7, − 3.5	0.017
New discharge to nursing care (2016–2018)	− 47.5	− 71.7, − 2.7	0.043
New discharge to rehabilitation	8.4	4.0,13.0	0.002
Returned back home	− 2.9	− 5.1, − 0.7	0.016
*New antiresorptive therapy by discharge*	1.9	− 0.7, 4.5	0.113

### Trends in Post-operative Outcomes

**Fig. 3:**
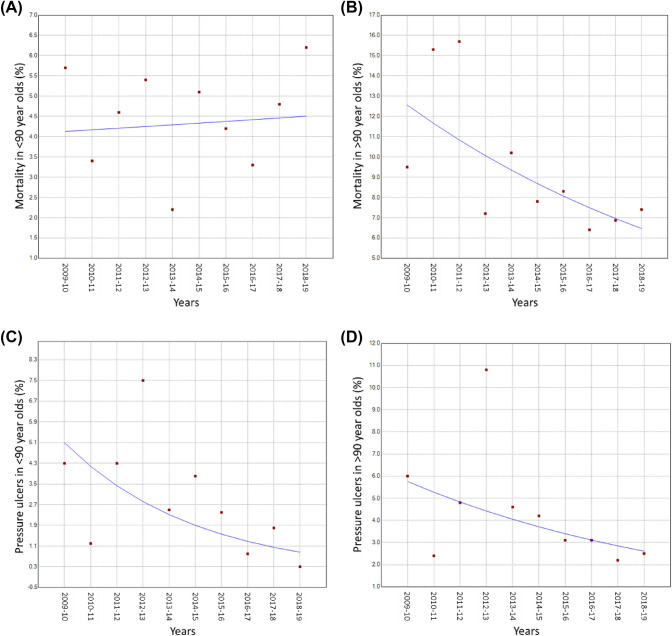
Temporal trends in-hospital mortality in patients < 90 years (**A**) and > 90 years (**B**); and pressure ulcers in patients < 90 years (**C**) and > 90 years (**D**) after hip surgery

There were no significant changes in failure to mobilise within one day of hip surgery. Table 2 shows that although there was an overall decline in the rates of pressure ulcers and mortality, this did not reach a significance level (Fig. 3A and B). However, when analysed separately by age groups (by upper quartile threshold: 90 years), there was a significant decline in pressure ulcers amongst patients under 90 years old: APC =  − 17.9 (95% CI − 32.7, 0.0, *P* = 0.050) and also a significant decline in mortality amongst those over 90 years old: APC =  − 7.1 (95% CI − 12.6, − 1.3, *P* = 0.024) (Fig. 3C and D).

**Fig. 4:**
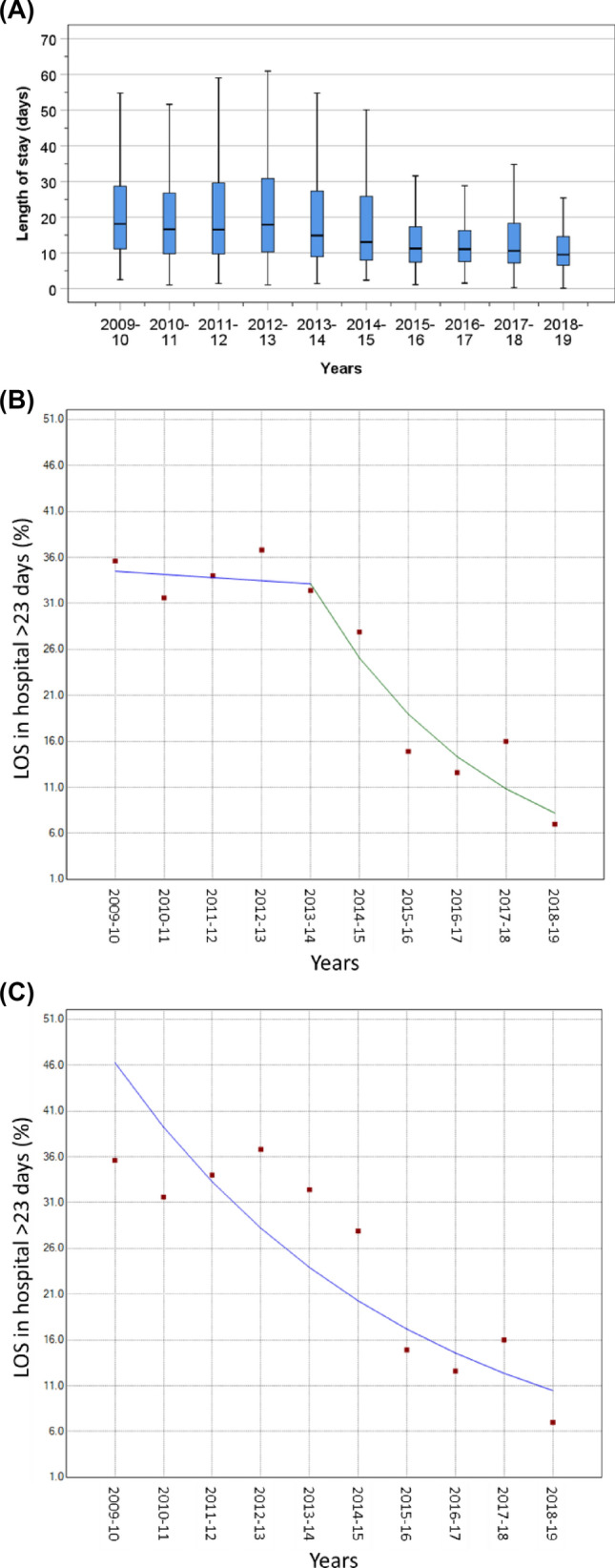
Median (interquartile range) length of stay in hospital (**A**) and proportions of patients who stayed longer than 23 days in hospital with a single join point (**B**) or with no join point (**C**)

The median LOS in hospital in the first two years (2009–2011) was just over 15 days which reduced relatively little until a high reduction from 2013 to 2019 (Fig. 4A). There was a single join point at 2013 that coincided with data collection of the new service (see Introduction) (Fig. 4B); the APC did not change between 2009 and 2013, whilst there was a significant decline from 2013 to the end of the study period (APC =  − 24.6, 95% CI − 31.2, − 17.4, *P* < 0.001) (Table 2). When no join point was used, the decline of the AAPC (= − 15.2, 95% CI − 21.1, − 8.8, *P* = 0.001) remained significant (Fig. 4C).

### Trends in Discharge

**Fig. 5:**
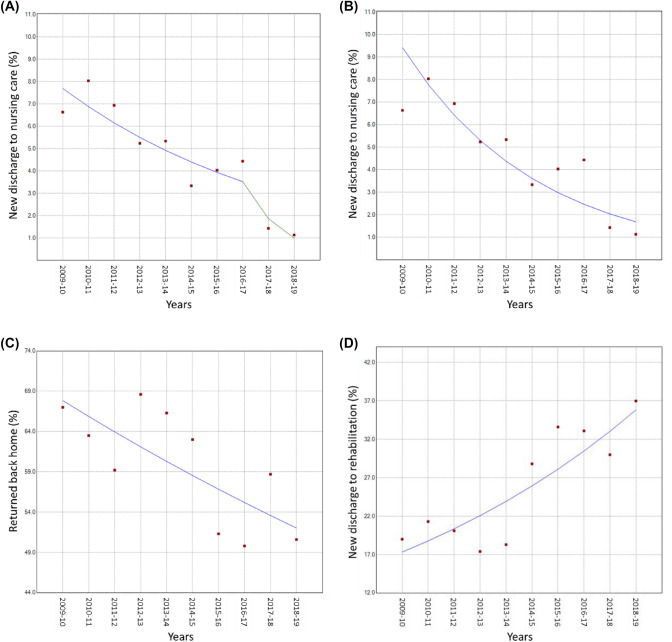
New discharge to nursing care with a single join point (**A**) and no join point (**B**), and returned back home (**C**) or new discharge to rehabilitation (**D**) amongst patients who were admitted from their own home

There was a single join point at 2016, coinciding with introduction of the COW model (see Introduction) (Fig. 5A); there was a moderate APC between 2009 and 2016 (APC =  − 10.6, 95% CI − 17.2, − 2.7, *P* = 0.017), followed by a sharper decline between 2016 and the end of the study period (APC =  − 47.5, 95% CI − 71.7, − 2.7, *P* = 0.043). The AAPC was − 17.5 (95% CI − 24.3, − 10.1, *P* = 0.001) (Fig. 5B).

There was a continuously decreasing trend in the proportions of patients returning home (APC =  − 2.9, 95% CI − 5.1, − 0.7, *P* = 0.016) (Fig. 5C), whilst the trend in new discharge to rehabilitation increased (APC = 8.4, 95% CI 4.0, 13.0; *P* = 0.002) (Fig. 5D).

**Fig. 6:**
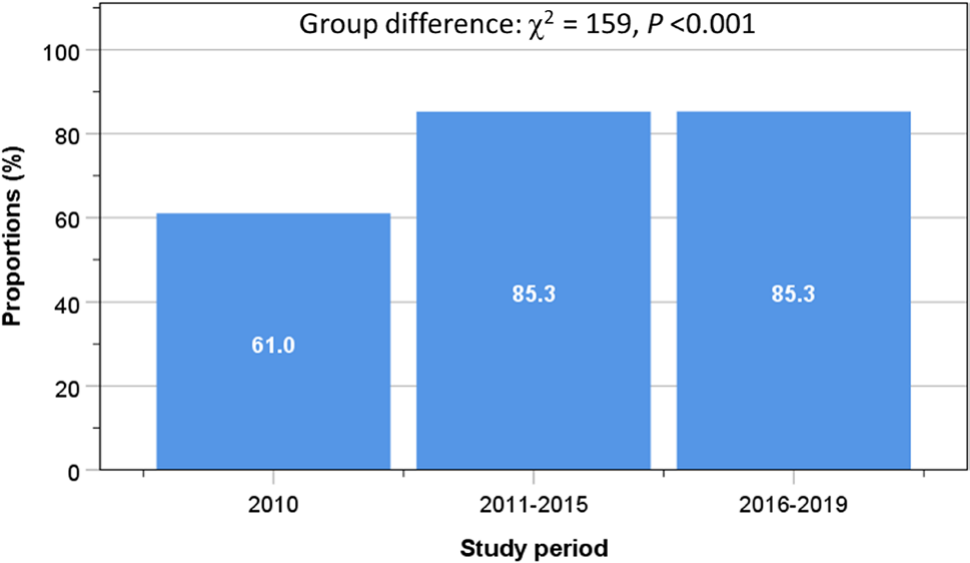
Proportions of patients receiving anti-resorptive agents according to the milestones of service development

The proportion of patients newly prescribed with an anti-resorptive agent was only 61% in 2010 when two orthogeriatricians were appointed later in that year (September 2010), rising significantly to 85.3% since 2011 (group difference: *χ*^2^ = 159, *P* < 0.001) (Fig. 6).

## Discussion

This study showed that after establishing the orthogeriatric service, there was a rapid reduction in elapsed time to surgery. This change was associated with improvement in a number of outcomes, most notably reduction in mortality amongst the oldest age group; incidence of pressure ulcers in the younger group; prolonged LOS in hospital; and new discharge to nursing care. Although people undergoing hip operations were increasingly older, and proportionally more of those over 90 years had severe systemic disease (ASA grade ≥ 3), there was no evidence for this impacting on detrimental effects including mortality and pressure ulcers. Our findings support the value of orthogeriatric service in caring for patients admitted to hospital with a hip fracture.

A number of factors may have influenced the temporal trends of outcomes observed in the present study. These include patient characteristics (their underlying health status), management aspects of patients from surgical and medical teams and reorganisation of the work system. The present study showed that patients undergoing hip surgery were increasingly older and had poorer physical status over time; therefore, the improvement in outcomes points towards changes in surgical and medical management throughout the period of study. The integrated care of a surgical and multidisciplinary orthogeriatric service thus plays a pivotal role in the improvement of patient outcomes observed in the present study. We found a clear benefit of reduction in delay in surgery (elapsed time > 36 h) including a reduction in mortality in older patients, pressure ulcers in younger patients, LOS and discharge to nursing care. Our findings were strengthened by further analysis showing that the rate of mortality was lower (5.1%) for elapsed time < 36 h compared with that of elapsed time ≥ 36 h (8.1%, *P* = 0.001).

An integrated orthogeriatric service is therefore likely to play a key role in the improvement of outcomes. An earlier retrospective study of 951 patients in Australia showed that orthopaedic–geriatric joint care of hip fractures was associated with reductions in postoperative complications and mortality [19: 12]. This is supported by a two-year study of 1894 patients showing a change in service from geriatric consultation model of care to an integrated orthogeriatric model led to reductions in LOS, time to surgery and mortality [13]. More recently, a large study of 58000 patients from 828 hospital in Germany found that a multidisciplinary orthogeriatric approach was associated with lower mortality [16]. We observed that although the rates of mortality were higher in older age, there was a significant trend in reduction in mortality amongst those over 90 years.

The joinpoint regression analysis of our observational study cannot establish causal relations. Indeed, different outcome measures appear to behave differently following changes in the orthogeriatric service. This is likely to be due to a time lag, some outcomes may change immediately such as time to surgery, whilst others transition more gradually. Most outcomes followed a progressive improvement whilst there was a rebound in time to surgery within 36 h after the initial rapid improvement, but the rise remained well below the peak. The early rapid decline occurred when our orthogeriatricians first arrived, encouraging orthopaedic surgeons to follow the Best Practice Tariff recommendation of time to surgery within 36 h. There may be a number of explanations for a rise after the first two years including more complex patients (older with high ASA score) being treated, who needed a longer period of stabilisation. Another factor may be due to a decline of the initial enthusiasm in response to new service changes, or there might have been additional changes to orthopaedic personnel (we do not have this information).

There was evidence that surgical techniques have changed over the time-frame of this study. Although arthroplasty remained the technique of choice, this technique had not changed over the years. By contrast, there has been a significant increase in the use of IMN which was mirrored by a decrease in SHS. Studies have shown that IMN was associated with lower risk of transfusion than SHS, but no differences in mortality between these two treatment techniques were demonstrated [24, 25]. We observed similar findings for the rates between arthroplasty, IMN, SHS for mortality: 5.4, 7.1 and 5.3% (*χ*^2^ = 3.7, *P* = 0.155) and for pressure ulcers: 3.5, 3.1 and 4.3 (*χ*^2^ = 2.1, *P* = 0.349).

Although the rates of hip fracture have declined or remained stable in many high-income countries including the UK [1, 5, 26, 27], the total numbers are high due to increased life expectancy [28]. Our observation of increasingly more hip operations performed for older age patients with high ASA scores may reflect the wider extension of operations to include patients with a high health risk and frailty. The introduction of the Best Practice Tariff may have served as the stimulus. In addition, the increasing age of patients admitted with a hip fracture [29] may have driven the need for operations amongst such a group. It is surprising that temporal trends of age at operation have rarely been reported. We found a significant increase in the number of patients over 90 years receiving surgical treatment. By contrast, a Danish study of patients with a similar age profile to those in this study found no change in age at operation between 1997 and 2017 [30]. However, a US study found an increase in mean age over the period 1991 and 2008 [31].

We observed hospital LOS shortened progressively, and the proportions of prolonged LOS (> 23 days) were reduced, over the period of study. These findings are consistent with previous observations in the USA for patients admitted with a hip fracture [30–32]. These changes may be due to increased capacity for community care, such as rehabilitation. This is supported by evidence from our study showing a progressive trend in new discharge to rehabilitation after a hip operation. Our findings were similar to those observed in previous studies [32]. A reduction in hospital LOS would reduce the financial costs as well as risk of hospital-acquired complications such as infection and pressure ulcers. The expansion of rehabilitation was also mirrored by a significant reduction in new discharge to nursing care. We found no changes in early readmissions, whilst other studies have reported increased readmission rates [31].

On admission, only 5% of patients had an antiresorptive agent. This lack of treatment is similar to observations in previous studies [33]. By discharge, 83% of our patients were prescribed with a new antiresorptive agent. We also observed that in the first year of study in 2010 (the first nine months were without orthogeriatric service), there were only 61% of patients newly prescribed with an anti-resorptive agents, rising to over 85% since 2011, which coincides with the initiation of our orthogeriatric service. There is evidence that the UK has done poorly with initiation of secondary prevention compared to other European countries [34]. Antiresorptive therapy after a hip fracture is important to prevent the risk of recurrent hip fractures [35] and lower the risk of death [36, 37].

There exists a number of orthogeriatric care models [14, 15, 38] including (1) *Orthopaedic ward and geriatric consultant service*; where patients are treated in the orthopaedic ward until discharged home or transferred to a rehabilitation centre. The geriatric consultation is only provided on request. This model is the simplest and was used by the orthopaedic team at our centre prior the establishment of orthogeriatric service. (2) *Orthopaedic ward and daily consultative service*: a variation of the traditional team where the geriatrician consults from admission to discharge. (3) G*eriatric and rehabilitation ward and orthopaedic consultant service*: patients are admitted to geriatric ward until discharge, whilst the orthopaedic surgeon is consultative. This model was used by our centre between 2010 and 2016. (4) *Orthopaedic ward and integrated care*: the orthopaedic surgeon and the geriatrician manage the patient together from admission until discharge. The patients are cared for on an orthopaedic ward, and the geriatrician is integrated into the orthopaedic team, working with a multidisciplinary team, and standardised treatment pathways are implemented. The COW model employed at our centre from 2016 is identical to the fourth model, except that patients are cared for on an orthogeriatric ward. It is recognised that these models may yield different outcomes [14]. Our findings suggest that improvement of outcomes was associated with the higher level of this integrated care model and is consistent with findings from meta-analyses of randomised controlled trials [14, 38].

### Strengths and Limitations

Our data were collected according to protocol outlined by the NHFD audit programme [19] which could conveniently be used to compare those in the national reports. Information in all patients admitted with a hip fracture were recorded in detail from the time of admission to discharge. Although this is a single-centred study, the patients’ characteristics are similar to patients admitted with a hip fractures in other areas of the UK [20].

In conclusion, the establishment of an orthogeriatric service was associated with a rapid reduction of elapsed time to hip surgery beyond 36 h, and surgery in more high-risk adults and a decline in prolonged LOS and discharge to nursing care, whilst without detrimental impact on mortality or prevalence of pressure ulcers.

## Supplementary Information

Below is the link to the electronic supplementary material.

Supplementary file1 (DOCX 523 KB)
