# Supplementary material for: Changes in Characteristics and Outcomes of Patients Undergoing Surgery for Hip Fractures Following the Initiation of Orthogeriatric Service: Temporal Trend Analysis
Source: Calcif Tissue Int. 2021 Aug 27;110(2):185–95. doi: 10.1007/s00223-021-00906-4 (PMC8784364; doi:10.1007/s00223-021-00906-4)

**SUPPLEMENTARY MATERIAL**

**Figure 1.** Annual mean (95% CI) age at time of hip surgery.


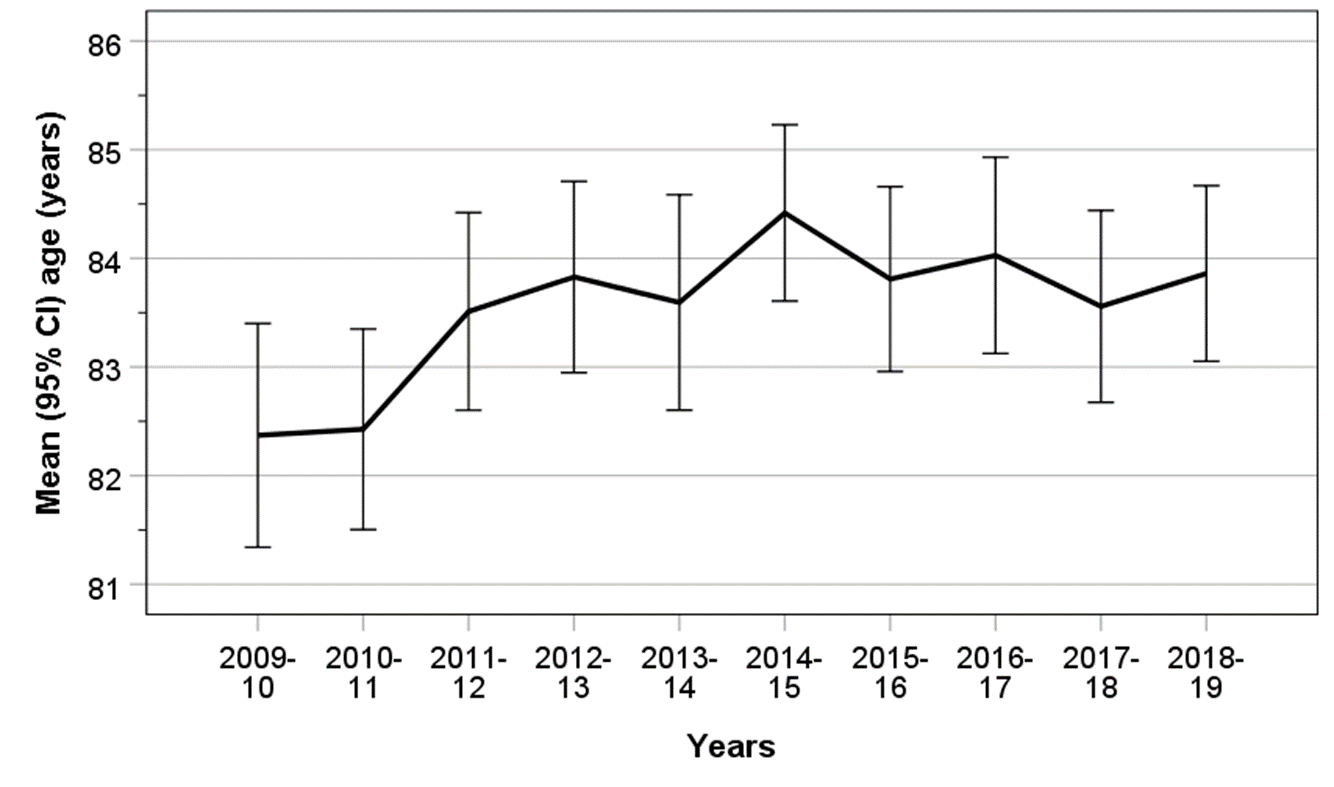


**Figure 2.** Trends in types of hip surgery: arthroplasty (**A**), IMN (**B**) and SHS (**C**).

(**A**)


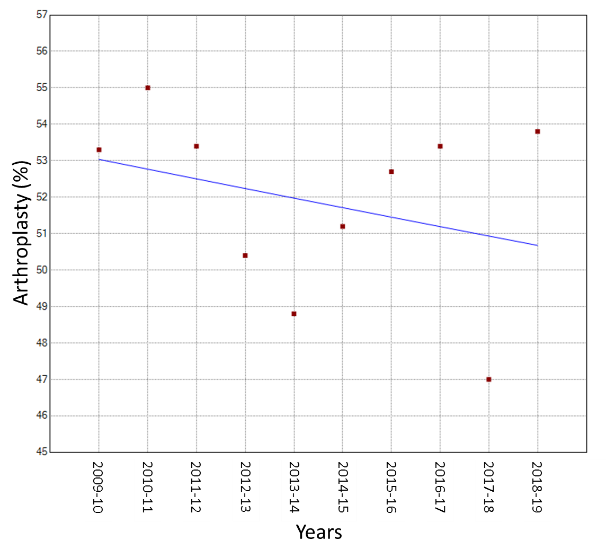


(**B**)


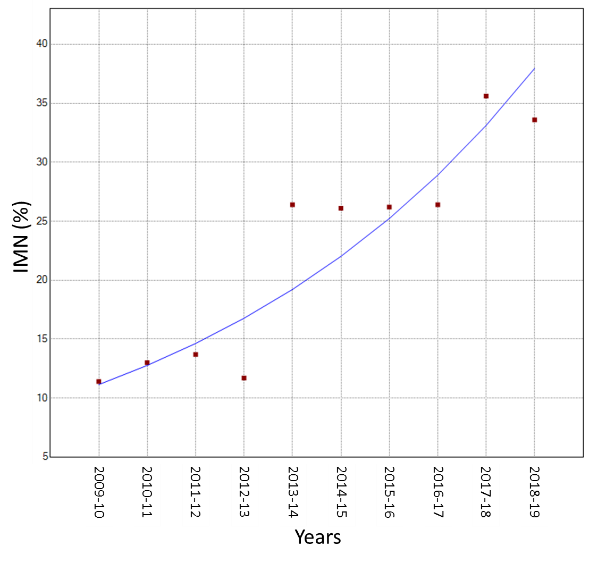


(**C**)


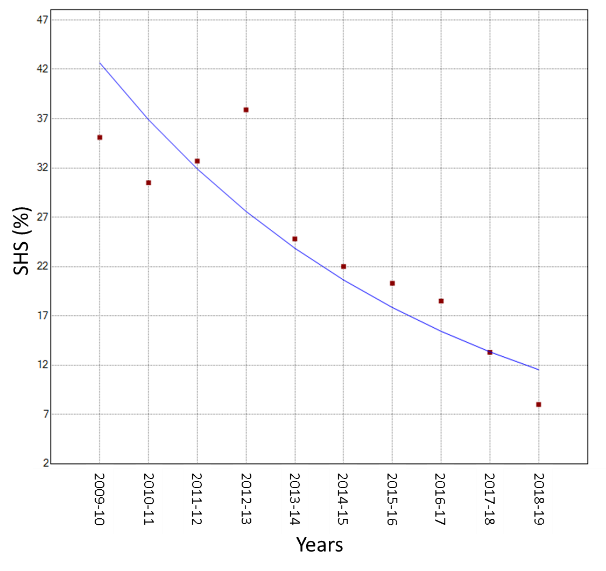


**Figure 3.** Mortality (**A**) and pressure ulcers (**B**) in hospital after hip surgery.

(**A**)


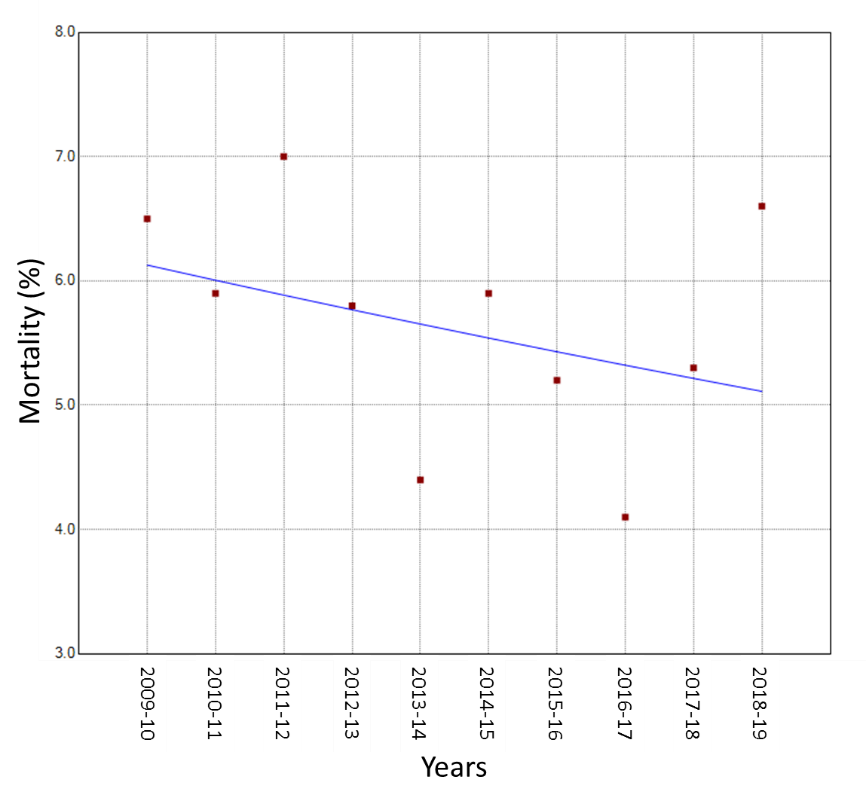


(**B**)


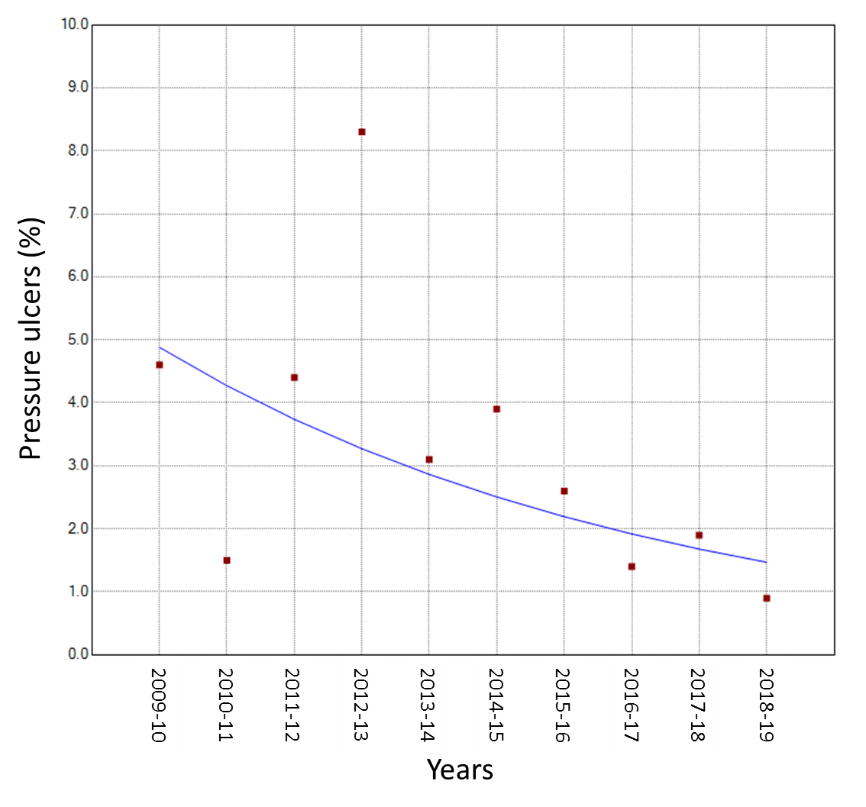

Supplement: Supplementary file 1 — Supplementary file1 (DOCX 523 KB) [file 223_2021_906_MOESM1_ESM.docx]
